# Comparative effectiveness of standard vs. AI-assisted PET/CT reading workflow for pre-treatment lymphoma staging: a multi-institutional reader study evaluation

**DOI:** 10.3389/fnume.2023.1327186

**Published:** 2024-01-11

**Authors:** Russell Frood, Julien M. Y. Willaime, Brad Miles, Greg Chambers, H’ssein Al-Chalabi, Tamir Ali, Natasha Hougham, Naomi Brooks, George Petrides, Matthew Naylor, Daniel Ward, Tom Sulkin, Richard Chaytor, Peter Strouhal, Chirag Patel, Andrew F. Scarsbrook

**Affiliations:** ^1^ Department of Radiology, Leeds Teaching Hospitals NHS Trust, Leeds, United Kingdom; ^2^ Leeds Institute of Health Research, University of Leeds, Leeds, United Kingdom; ^3^ Mirada Medical Ltd., Oxford, United Kingdom; ^4^ Alliance Medical Ltd., Warwick, United Kingdom; ^5^ Department of Radiology, York and Scarborough Teaching Hospitals NHS Foundation Trust, York, United Kingdom; ^6^ Department of Radiology, Newcastle upon Tyne Hospitals NHS Foundation Trust, Newcastle, United Kingdom; ^7^ Department of Radiology, Royal Cornwall Hospitals NHS Trust, Truro, United Kingdom

**Keywords:** artificial intelligence, lymphoma, PET/CT, multi-reader study, efficiency

## Abstract

**Background:**

Fluorine-18 fluorodeoxyglucose (FDG)-positron emission tomography/computed tomography (PET/CT) is widely used for staging high-grade lymphoma, with the time to evaluate such studies varying depending on the complexity of the case. Integrating artificial intelligence (AI) within the reporting workflow has the potential to improve quality and efficiency. The aims of the present study were to evaluate the influence of an integrated research prototype segmentation tool implemented within diagnostic PET/CT reading software on the speed and quality of reporting with variable levels of experience, and to assess the effect of the AI-assisted workflow on reader confidence and whether this tool influenced reporting behaviour.

**Methods:**

Nine blinded reporters (three trainees, three junior consultants and three senior consultants) from three UK centres participated in a two-part reader study. A total of 15 lymphoma staging PET/CT scans were evaluated twice: first, using a standard PET/CT reporting workflow; then, after a 6-week gap, with AI assistance incorporating pre-segmentation of disease sites within the reading software. An even split of PET/CT segmentations with gold standard (GS), false-positive (FP) over-contour or false-negative (FN) under-contour were provided. The read duration was calculated using file logs, while the report quality was independently assessed by two radiologists with >15 years of experience. Confidence in AI assistance and identification of disease was assessed via online questionnaires for each case.

**Results:**

There was a significant decrease in time between non-AI and AI-assisted reads (median 15.0 vs. 13.3 min, *p* < 0.001). Sub-analysis confirmed this was true for both junior (14.5 vs. 12.7 min, *p* = 0.03) and senior consultants (15.1 vs. 12.2 min, *p* = 0.03) but not for trainees (18.1 vs. 18.0 min, *p* = 0.2). There was no significant difference between report quality between reads. AI assistance provided a significant increase in confidence of disease identification (*p* < 0.001). This held true when splitting the data into FN, GS and FP. In 19/88 cases, participants did not identify either FP (31.8%) or FN (11.4%) segmentations. This was significantly greater for trainees (13/30, 43.3%) than for junior (3/28, 10.7%, *p* = 0.05) and senior consultants (3/30, 10.0%, *p* = 0.05).

**Conclusions:**

The study findings indicate that an AI-assisted workflow achieves comparable performance to humans, demonstrating a marginal enhancement in reporting speed. Less experienced readers were more influenced by segmentation errors. An AI-assisted PET/CT reading workflow has the potential to increase reporting efficiency without adversely affecting quality, which could reduce costs and report turnaround times. These preliminary findings need to be confirmed in larger studies.

## Introduction

There is an increasing incidence of lymphoma worldwide, with 544,352 new cases of non-Hodgkin lymphoma globally in 2020 and 83,087 cases of Hodgkin lymphoma; there was a higher occurrence in high-income countries and greater mortality rates in low-income countries (1, 2). Fluorine-18 fluorodeoxyglucose (FDG)-positron emission tomography/computed tomography (PET/CT) has become the gold standard imaging technique for staging and response assessment of high-grade lymphoma (3). The Lugano staging classification is the most used clinically (Table 1) (4).

**Table 1:** Lugano staging classification.

Stage	Description
Stage 1	One nodal group
Stage 1E	Single site of extra-nodal involvement in the absence of nodal involvement
Stage 2	Two or more nodal groups on a single side of the diaphragm
Stage 2E	Contiguous extra-nodal involvement from a nodal site with all disease confined to a single side of the diaphragm
Stage 3	Nodal involvement on both sides of the diaphragm (the spleen is regarded as nodal involvement)
Stage 4	Involvement of one or more extra-nodal organs or tissue beyond the designated E stages

A suffix of A or B can be added to the staging classification to denote the presence or absence, respectively, of B-symptoms. B-symptoms are described as the presence of fevers, night sweats or the loss of 10% of body weight.

Given the wide variation in the extent of lymphomatous disease at presentation, with imaging appearances sometimes being confounded by physiological FDG uptake or secondary pathology (5), the time taken to report a staging lymphoma PET/CT varies and can be time consuming in more complex cases. There is currently a global workforce crisis in radiology, with the Royal College of Radiologists in the UK recently reporting a 29% shortfall in consultant radiologists, which is expected to increase to 36% by 2026 (6, 7). This is due to a combination of rising imaging demand in an aging population, professional burnout and finite training positions available for radiologists. Artificial intelligence (AI) may offer support to the current workforce and help meet rising imaging demands with studies demonstrating the feasibility of algorithms to tackle a number of image-processing tasks applied to PET including image denoising of low count images (8, 9), image enhancement (10, 11), lesion detection and segmentation (12, 13). For instance, Sibille et al. (12) proposed a convolutional neural network (CNN) to detect and classify FDG-positive uptake regions on PET/CT and predict anatomical location in lymphoma and lung cancer with an area under the curve (AUC) performance above 0.95. Similarly, Weisman et al. (13) used an ensemble of 3D CNNs (DeepMedic) implemented to detect nodal involvement in lymphoma with a reported detection performance comparable to that of experienced nuclear medicine physicians.

In a previous study, we reported the technical performance of a deep learning model, consisting of an ensemble of patch-based 3D DenseNet (14), for the detection and quantification of lymphomatous disease on FDG PET/CT (15). The model trained on 300 PET/CT cases achieved a sensitivity of 86%, three false positives (FP) and a true-positive/FP ratio of 1.69 per scan, while a per-voxel analysis yielded a sensitivity of 93%, positive predictive value (PPV) of 88% and DICE score of 86%. However, when designing a clinical tool to aid in reporting, it is necessary to not only look at the effect of the tool on technical performance metrics such as sensitivity and specificity, but also on the efficiency and accuracy of scan interpretation, whether these vary between different sub-groups of users and if the tool has an influence (negative or positive) on reporting behaviour (16). It is also important to sample a real-world spectrum of disease to identify the variation in the performance of the AI tool between groups of cases (17).

The aim of the present study was to assess the feasibility of an AI-assisted PET/CT reporting workflow for pre-treatment staging in high-grade lymphoma. The influence of this tool on the speed and quality of reporting by readers with variable levels of experience was measured within a research environment emulating standard PET/CT clinical reporting conditions using a real-world caseload with and without AI-contouring of disease. The dataset also included cases with deliberately imperfect contouring of disease to assess whether readers might be biased by AI technology, the results of which will inform larger future studies.

## Methods

### Study design

**Figure 1:**
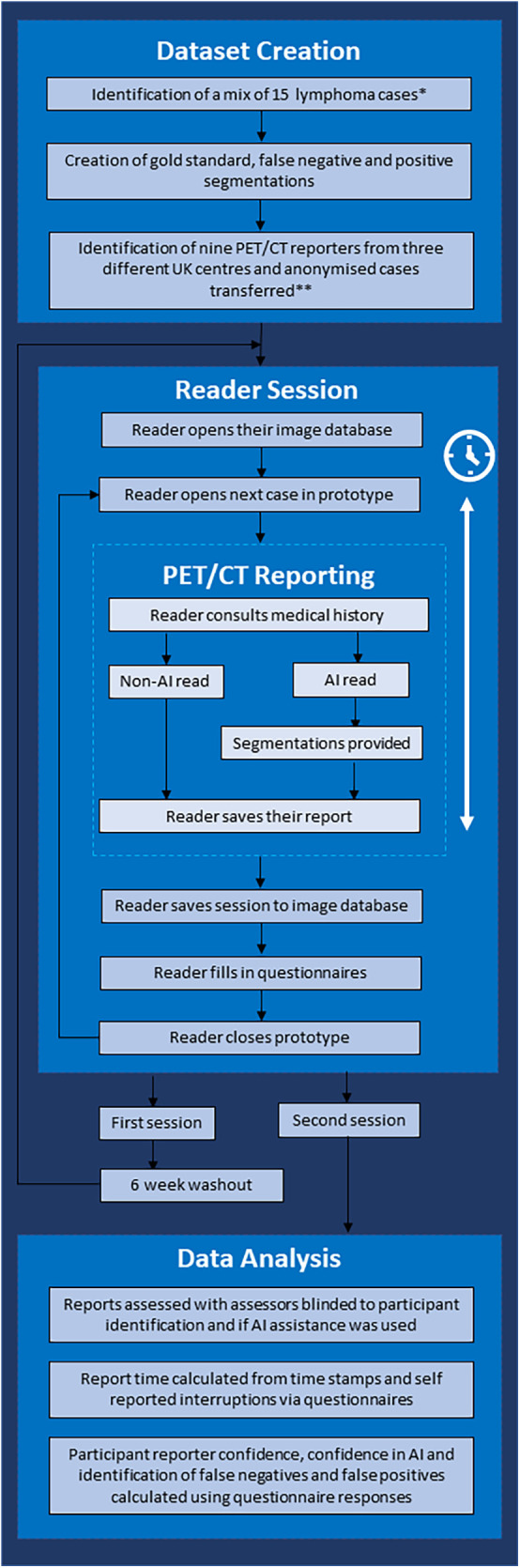
Flow chart demonstrating the study workflow. *Cases demonstrating metformin bowel uptake, brown fat uptake and a mix of stage II and stage IV cases were included. **Three senior consultants (>5 years of consultant experience), three junior consultants (<5 years of experience) and three trainees were included.

The study used a crossover design to compare an AI-assisted PET/CT reading and reporting workflow (Intervention) to a standard of care clinical reporting workflow (Comparator) (Figure 1). The objective of this study was to demonstrate whether there was an improvement in speed and/or quality of reporting with the Intervention.

### Ethics

Informed written consent was obtained prospectively from all patients at the time of imaging for use of their anonymised FDG PET/CT images in research and service development projects. As this was a retrospective study, not involving patient contact or alteration of treatment, after discussion with the institutional Research and Innovation Department where scans were performed, it was agreed that this represented a service improvement project and formal approval from the ethics committee was waived.

### Participants

#### Patient characteristics

In total, 15 high-grade lymphoma staging FDG PET/CT studies (13 Hodgkin lymphoma and 2 diffuse large B cell lymphoma) were retrospectively selected from cases performed at a single large tertiary centre between January 2008 and January 2020. The imaging dataset included PET/CT studies from 11 women and 4 men with a mean age of 39.8 years (range 18–67) and represented a real-world spectrum of disease extent. Only adult studies were included, and all the imaging was performed as part of routine clinical practice.

#### PET/CT imaging

Patients fasted for 6 h before the administration of intravenous fluorine-18 FDG (4 MBq/kg). The breakdown of the PET acquisition parameters used is provided in Table 2. Low-dose unenhanced diagnostic CT was used for attenuation correction with the following parameters: slice thickness 3.75 mm; pitch 6; 140 kV; and 80 mAs.

**Table 2:** Reconstruction parameters for the scanners used.

Scanner	No. PET/CT studies (type of AI segmentation)	Matrix	Voxel size [column, row, slice thickness (mm)]	Reconstruction	Scatter correction	Randoms correction
GE Healthcare STE	2 (1 FP, 1 FN)	128	4.6875 × 4.6875 × 3.27	OSEM	Convolution subtraction	Singles
GE Healthcare Discovery 690	5 (1 FP, 1 FN, 3 GS)	192	3.65 × 3.65 × 3.27	VPFX	Model based	Singles
GE Healthcare Discovery 710	4 (1 FP, 2 FN, 1 GS)	192	3.65 × 3.65 × 3.27	VPFX	Model based	Singles
Philips Gemini TF64	4 (1 FN, 2 FP, 1 GS)	144 or 169	4 × 4 × 4	BLOB-OS-TF	SS-Simul	DLYD

DLYD, delayed event subtraction; OSEM, ordered subsets expectation maximisation; SS-simul, single-scatter simulation; VPFX, Vue point FX (3D time of flight); BLOB-OS-TF, a 3D ordered subset iterative time of flight reconstruction algorithm (spherically symmetric basis function ordered subset); FN, false negative; FP, false positive; GS, gold standard.

#### Disease presentation

A representative cross-section of lymphomatous disease was selected. In addition, PET/CT studies included scenarios encountered in routine clinical practice including metformin-related bowel uptake, brown fat activity, stage II disease, stage IIE/stage IE and stage IV disease. Cases were carefully curated to provide a mix of complexity and a spread of expected reporting times. They also provided situations where disease may be under- or over-estimated, and in some cases could affect patient management.

#### Readers

Nine radiologists (all active PET/CT reporters) from three different UK institutions (Figure 1) participated in the reader study. Participants consisted of an equal split of trainee reporters (no independent reporting experience, *n* = 3), junior consultants (<5 years of consultant PET/CT reporting experience, *n* = 3) and senior consultants (>5 years of consultant PET/CT reporting experience, *n* = 3). Each reader had access to a training video and instructional document, which was available before and during reporting sessions. The participating radiologists were also given an opportunity to ask the research team questions before the reading sessions. The reporting environment was standardised for all readers and closely mimicked routine clinical workflow, but none had access to the AI-assisted tool before the second read.

#### Study infrastructure

The reader study was performed using an established clinical PET/CT reporting platform (Alliance Medical Ltd., UK) with anonymised cases loaded into a training version of the standard PACS software (InteleRad, Montreal, Canada). Cases were assigned to individual readers within PACS as per the normal clinical workflow and imaging was reviewed on the same workstations used in routine practice albeit using a research prototype version of the standard PET/CT visualisation software (XD; Mirada Medical, Oxford, UK). Clinical reports were dictated into the training PACS as per the standard of care. Screen recording software (FlashBack Express Recorder v5.53.0.4690; Blueberry Software, UK) was used to monitor user interactions, and software log files containing timestamps, reports and video recordings were collated for analysis.

#### Standard of care read

For the first reading session, participating radiologists were each assigned the same 15 PET/CT studies to evaluate and report as per their normal routine using the Alliance Medical PET/CT reporting platform and a research prototype built on Mirada XD configured with their usual user preferences. A reporting workstation was used at each institution by the participant radiologists to report PET/CT cases. The study was specifically designed to closely emulate the standard clinical reporting workflow within a monitored research environment.

#### AI-assisted read

**Figure 2:**
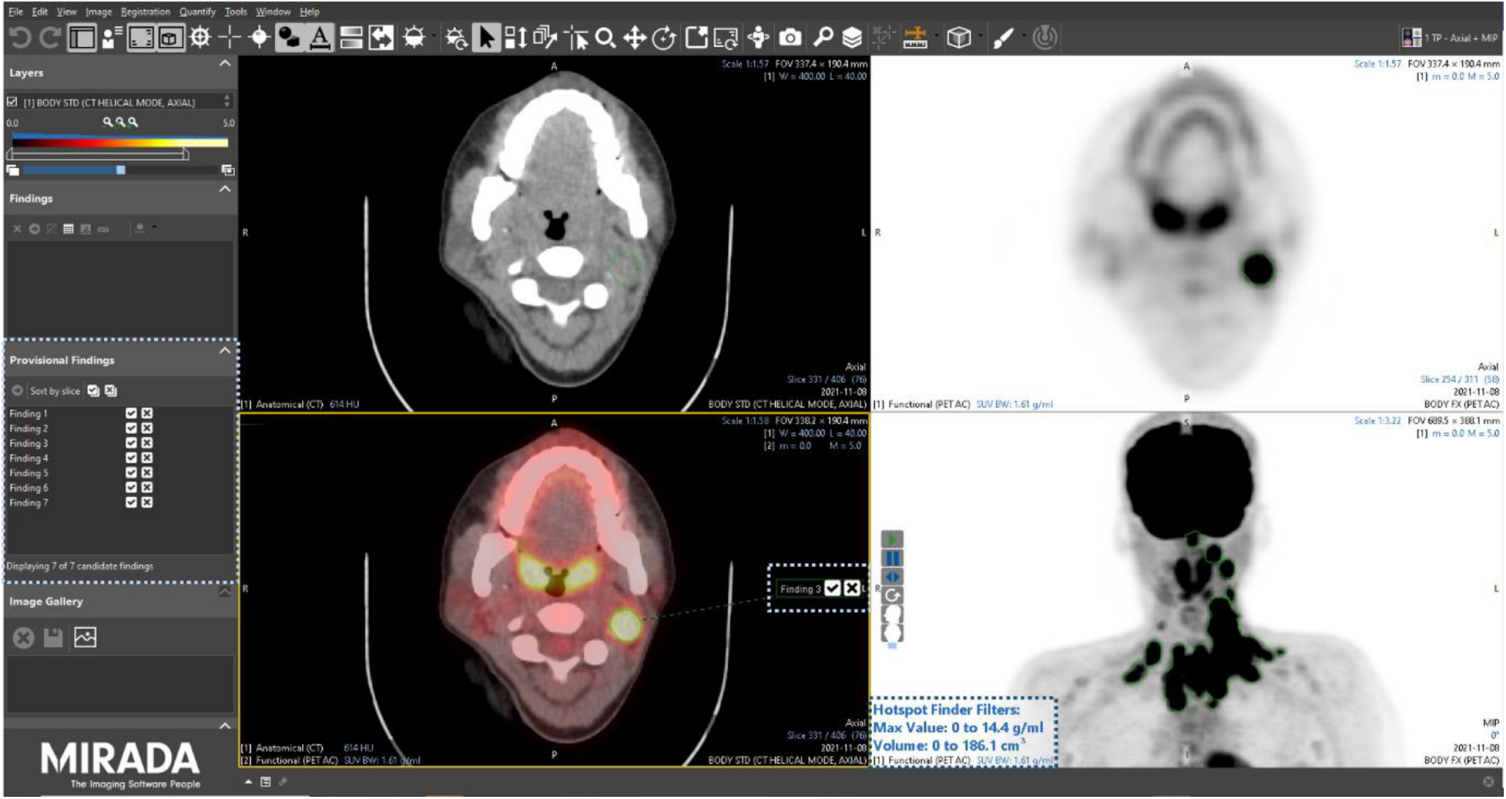
Screenshot of the Mirada Medical research prototype with the AI-assisted module enabled. Corresponding user interface elements are highlighted by dotted boxes.

After a washout period of 6 weeks, all readers were assigned the 15 PET/CT studies a second time, albeit in a different order, using the same Alliance Medical PET/CT reporting platform, the only difference being in this instance the PET/CT viewing software (Mirada XD research prototype) had an AI-assisted lesion detection and segmentation module enabled. The module combines a dedicated user interface (UI) with a curated list of candidate imaging findings that can be pre-computed using an AI model. The technical detail and results of AI models trained on this population of lymphoma patients have been reported elsewhere (15). When launching a PET/CT study in the research prototype, a companion tool automatically opened a list of candidate findings to assist the reader in their clinical PET/CT read and report (Figure 2). The use of the tools was not prescriptive, and participants were free to use the AI assistance as they wished.

Participants were blinded to the study design and outcome measurements (i.e., speed and quality of reporting) and were not specifically told that the same PET/CT images would be assigned to them (the same cases were assigned different ID numbers between the Intervention and Comparator arms of the study).

#### Candidate findings

**Figure 3:**
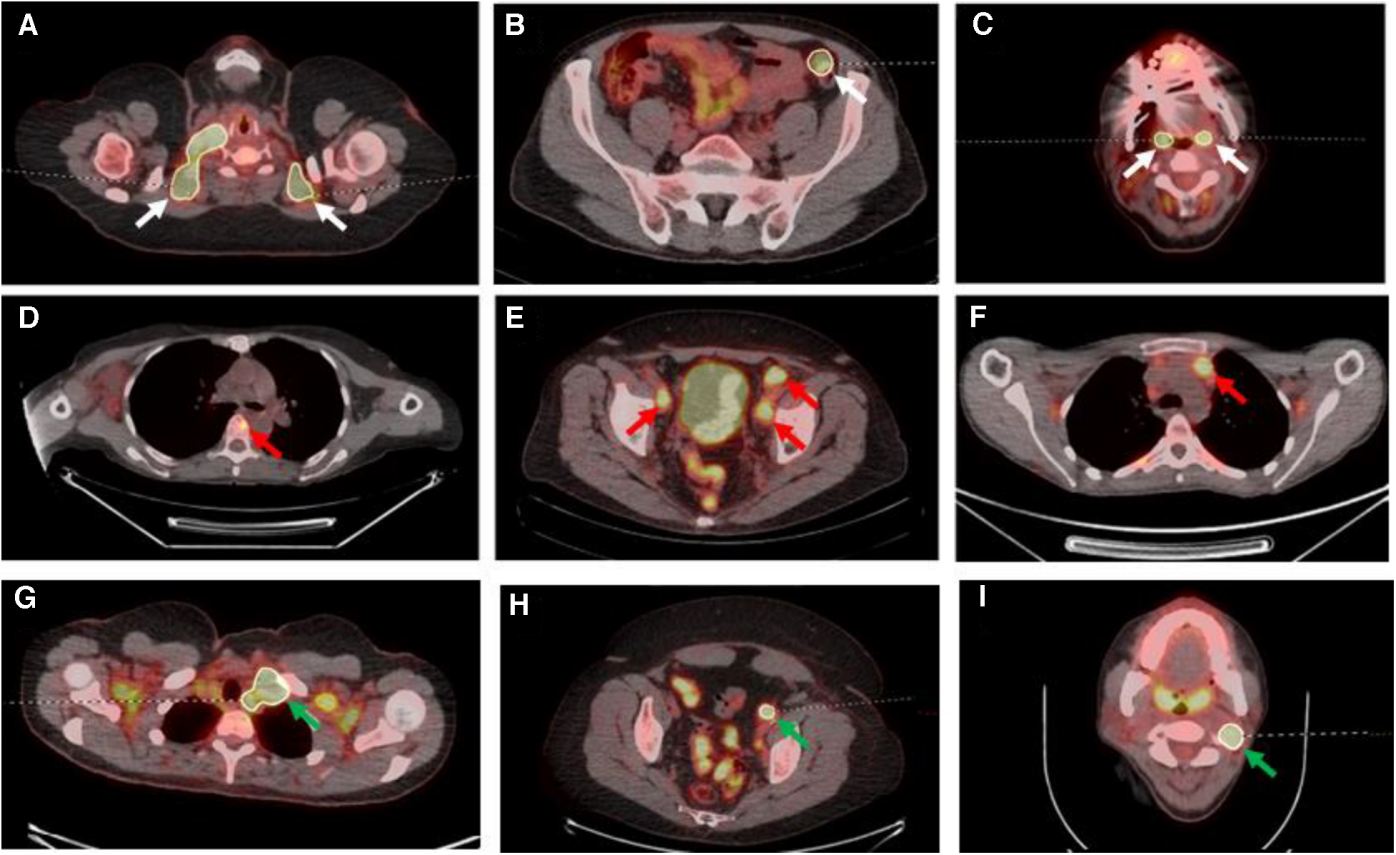
Select axial fused PET/CT slices depicted false positive segmentations (**A–C**), false negative segmentations (**D–F**) and gold standard (**G–I**). (**A**) Segmentations of brown fat; (**B**) the incorrect segmentation of the descending colon in a patient on metformin; (**C**) the segmentation of the palatine tonsils; (**D**) the missing segmentation of osseous involvement of a vertebral body (red arrow); (**E**) the missing segmentation of pelvic sidewall nodal disease (red arrows); (**F**) the missing segmentation of anterior mediastinal nodal disease (red arrow); (**G**) correct segmentation of left level IV lymph node (green arrow) on a background of brown fat uptake; (**H**) correct left external iliac lymph node (green arrow) segmentation on a background of metformin bowel uptake; (**I**) correct left level II lymph node segmentation (green arrow) on a background of physiological palatine tonsil uptake.

For the second (AI-assisted) read, all pre-segmentations had been performed by a radiologist with 7 years of experience and checked by a dual-certified radiologist and nuclear medicine physician with >15 years of experience in oncological PET/CT interpretation. The segmentations presented to the readers were clinically curated by the research team to correspond to the full ground-truth segmentation in five of the PET/CT studies (gold standard segmentation (GS)), included additional FP regions in another five of the PET/CT studies and deliberately omitted some regions of disease in another five PET/CTs (false negative (FN)) (Figure 3).

#### Speed of reporting

The read duration was measured as the time from opening the case in the prototype PET/CT reading software to signing off the report. A combination of timestamps from an analytics file, electronic timestamp on the report and screen recording files were utilised to permit accurate measurement of the time taken to report each case. Reporters were asked to log any interruptions to their reporting in the per-case questionnaire and this was accounted for in subsequent analyses.

#### Report quality

The participants’ report quality for each case was independently assessed by one of two dual-certified radiology and nuclear medicine physicians with >15 years of experience using a standard 5-point audit score used in routine clinical practice (18, 19) (Table 3). Reports generated in both arms of the study (with and without AI) by a participant radiologist for a specific PET/CT case were scored for quality by the same assessor.

**Table 3:** 5-point quality scoring system used within the Alliance Medical Ltd reporting network.

Score	Description
5	Perfect report
4	Trivial difference—no amendment needed
3	Minor disagreement—no clinical significance
2	Moderated disagreement—likely clinically significant
1	Major error—likely to lead to adverse outcomes

During the scoring sessions, each assessor was blinded to the identity of the participant radiologist, their level of experience and whether the report was prepared using AI assistance. The auditors reviewed reports in batches of 15 cases. These were presented to the assessors using a random mix of participants and sessions in each batch. Case IDs on the reports were anonymised, and the layout and structure of the reports were unified to have the same headings, i.e., ‘Clinical History’, ‘Technique’, ‘Findings’ and ‘Comments’. Due to the clinical history and technique sections being missing in some cases, all reports were presented to the assessor with the same details for these two sections to avoid bias.

#### Inter-observer variability

Approximately one-third of all reports were assessed by both assessors to allow inter-observer reliability to be calculated using Cohen's kappa, with a kappa of 0 equating to an agreement equivalent to chance and a kappa of 1 indicating perfect agreement (20).

#### Per-case questionnaire

After each case, participants were asked to complete an online questionnaire (https://www.sogolytics.com) regarding the findings of the PET/CT case, how confident the participant was in their report and if they had any interruptions during their read (Supplementary Material 1). For AI-assisted cases, participants were also asked how confident they were with the AI assistance capturing the extent of disease and if there was any disease missed or falsely added by the AI assistance tool.

#### Post-study survey

Participants were asked to complete an exit questionnaire on completion of both sessions that asked them about their experience of using the AI assistance and if they felt it influenced the speed of their reporting and the content of their reports (Supplementary Material 1).

### Statistical analysis

#### Speed of reporting

The overall time taken for each study between the first and second session was compared using a Wilcoxon signed-rank test (WSRT).

#### Quality of reporting

Significance was calculated using the McNemar–Bowker test for quality of reporting scores. Audit scores 4 and 5 were combined for analysis as these were considered to represent clinically acceptable reports.

#### Reporter confidence

The comparison between reporter confidence, reading the same image cases with the non-AI and AI-assisted workflows, was performed using the WSRT. In addition, the Mann–Whitney *U* test (MWUT) was used to compare reporter confidence when reading a distinct set of image cases associated with different types of segmentation (GS, FP, FN).

All statistics were performed using python (v3.8) with libraries scipy.stats (v1.9.3) and statsmodels.stats (v0.13.5). A *p*-value <0.05 was considered significant.

## Results

### Reporting time

**Figure 4:**
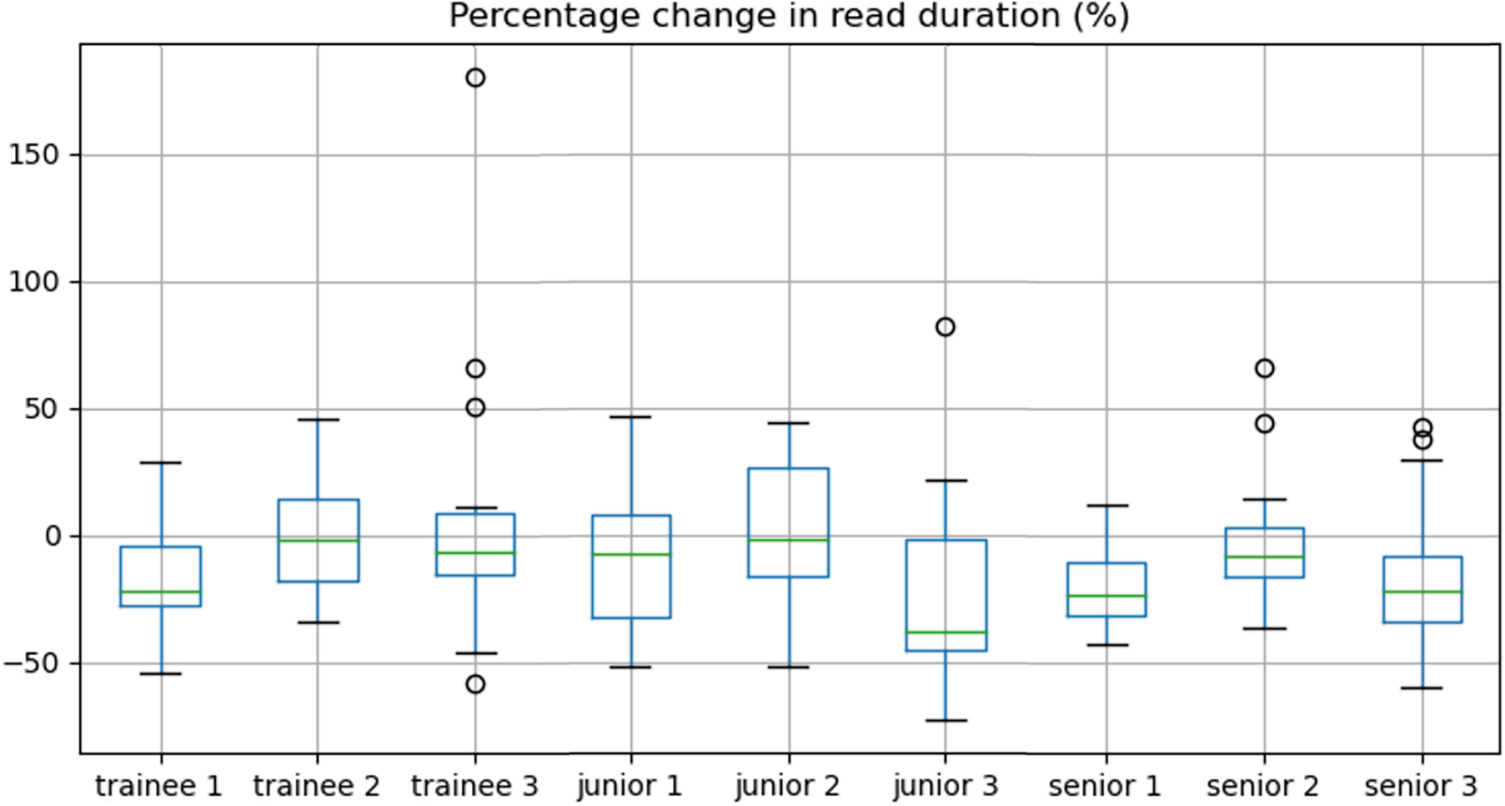
Box-and-whisker plots demonstrating the percentage change in read duration for each reader.

Overall, there was a significant decrease in reporting time between non-AI and the AI-assisted reads (median 15.0 vs. 13.3 min, IQR = (11.8–19.5 min) vs. (9.9–17.6 min), *p* < 0.001) representing a time reduction of 11.3%. Sub-analyses by reporter experience showed this held true for junior (median 14.5 vs. 12.7 min, time reduction of 12.4%, *p* = 0.03) and senior consultants (median 15.1 vs. 12.2 min, time reduction of 18.7%, *p* = 0.03) with no significant improvement in trainees (median 18.1 vs. 18.0 min, *p* = 0.2). The percentage change in reporting duration between the two reads is shown per participant radiologist in Figure 4.

### Report quality

There was no significant difference between the AI and non-AI-assisted reads (*p* = 0.11) when comparing the quality scores derived from the audited reports. This did not alter when stratifying data into trainee (*p* = 0.8), junior (*p* = 0.8) and senior reporters (*p* = 0.08), and by segmentation type, GS (*p* = 0.17), FP (*p* = 0.20) and FN (*p* = 0.51). The trainee quality scores increased in 4/45 cases, stayed the same in 37/45 cases and decreased in 4/45 cases between reads. The junior reporters scores increased in 1 of 45 cases, stayed the same in 40/45 cases and decreased in 4/45 cases between reads. Most of the quality scores stayed the same for the senior reporters (*n* = 44), with only one case decreasing between the reads.

### Ancillary analyses

#### Additional analyses on report quality

**Figure 5:**
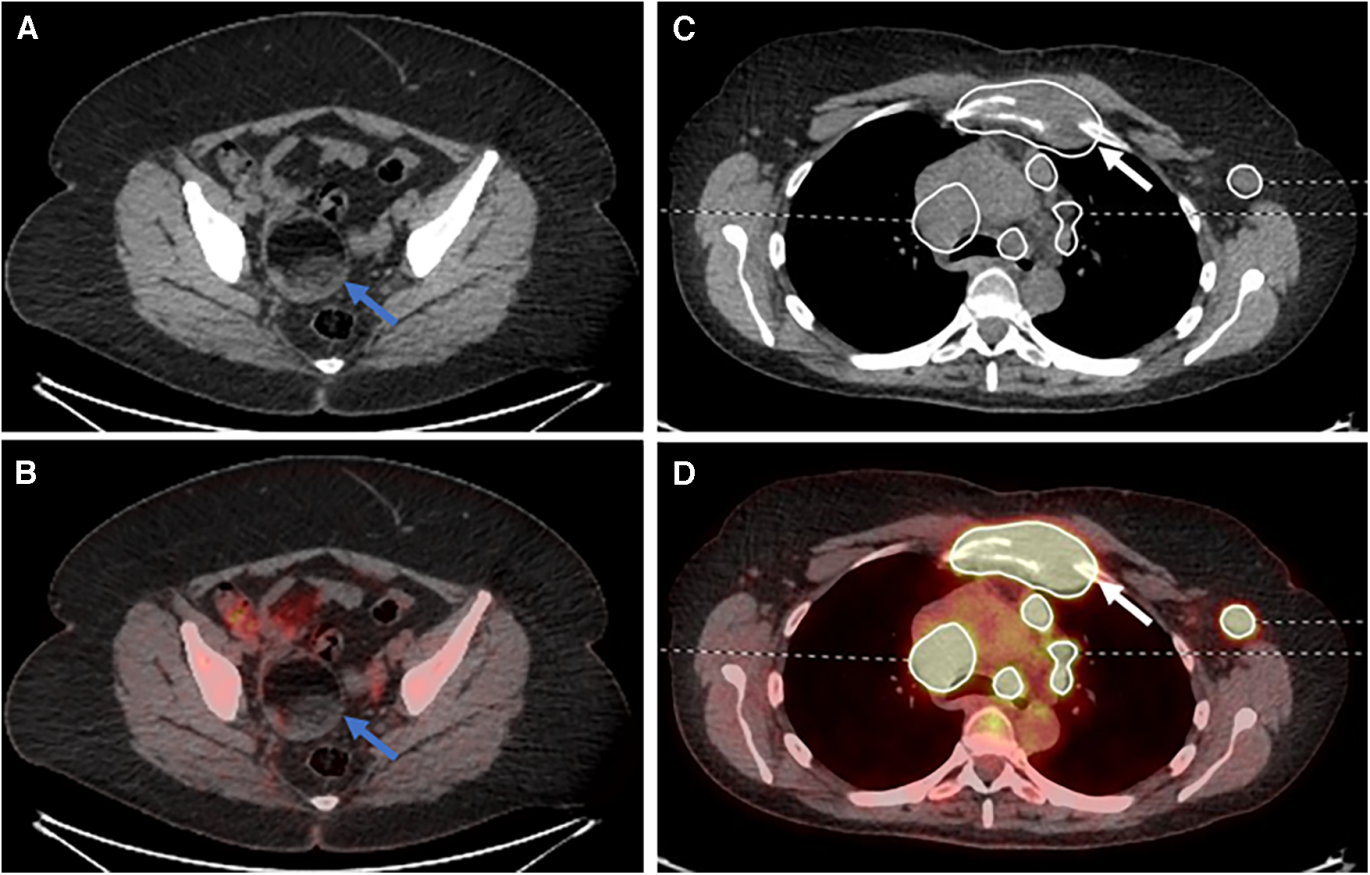
Select (**A**) axial CT and (**B**) fused PET/CT images demonstrating an ovarian dermoid cyst (blue arrows). Select (**C**) axial CT and (**D**) fused PET/CT images demonstrating destruction of the manubrium due to lymphomatous disease (white arrows).

A post-hoc analysis demonstrated a single lower quality score for a senior reporter in session 2 (AI-assisted read). This was a case where the reporter had inaccurately described the case as bulk disease on both the standard of care and AI-assisted reads. However, on the AI-assisted read, they also omitted mentioning an incidental ovarian dermoid (Figures 5A,B).

There were four cases of lower scores for the AI read when compared to the non-AI read for the junior consultant participants. The first was the mislabelling of physiological adnexal uptake within the pelvis as lymphomatous disease, although this was not segmented by the AI assistance tool. Two cases were due to the correct identification of all the disease but the readers mislabelling stage IIE as stage IV disease. The last case was the mislabelling of the severity of a pericardial effusion as being a shallow effusion.

There were four cases of lower scores for the AI read when compared to the non-AI read for the trainee participants: the first was the mislabelling of physiological adnexal uptake within the pelvis as lymphomatous disease, although this was not segmented by the AI assistance tool; the second was not mentioning the destruction of the manubrium, although the disease was provided as part of the segmentation (Figure 5C,D); the third was the misinterpretation of a pericardiac node as being below the diaphragm, changing the stage from stage II to stage III; and the fourth was the failure to mention the adnexal dermoid cyst (Figure 5A,B) and the reactive bone marrow uptake.

For the one case in the junior consultant cohort where the score improved between the non-AI-assisted read and the AI read, the reader had erroneously included potential uptake within the spleen, which changed the staging from stage IIE to stage IV disease. The AI-assisted segmentation for this case was correct in not labelling disease within the spleen. On the AI read, the report was staged correctly as stage IIE. For the four cases where the quality of reports improved in the trainee cohort, in the first case the participant reported an area of extra-nodal disease in the liver, which was not reported on the original non-AI read. The AI-assisted segmentation did highlight the extra-nodal disease in this case. Two reports included more areas of neck lymphadenopathy within the report on the AI-assisted read. The fourth report included the presence of bone involvement, which was not included on the original non-AI read.

### Inter-observer reliability

Of the 270 reports (9 reporters × 15 cases × 2 reads), 78 (29%) cases were assessed by both auditors. Of these cases that were assessed twice, the median audit quality score for both assessors was 5 with a mean score of 4.5 for assessor 1 and 4.4 for assessor 2. There was ‘substantial’ agreement between the two assessors, with a Cohen's kappa of 0.67.

### Reporter confidence

Two questionnaires were missing from the AI-assisted read for one junior consultant; therefore, only 14 cases were analysed for the FN and FP groups in the junior consultant cohort (5 cases × 3 junior consultants − 1 missing case).

There was a significant increase in confidence of disease identification with AI assistance (median 7/10 vs. 8/10, WSRT *p* < 0.001). This held true when splitting the data into FN, GS and FP. Participant confidence in AI accurately capturing disease significantly decreased between the GS and FN cohorts (median 8 vs. 6, MWUT *p* < 0.001), with no significant difference between the GS and FP cohorts.

Of the nine participants, six felt biased by auto-segmentations. However, in 80/88 (91%) cases, FPs/FNs did not adversely influence the report content compared to baseline. Trainees misinterpreted 2/15 (13%) FP findings vs. 2/14 (14%) for junior and 1/15 (7%) for senior consultants. Similarly, 3/15 (20%) FN segmentations were overlooked by trainees, but these pitfalls were spotted by all junior and senior consultants.

## Discussion

To the best of our knowledge, this is the first study demonstrating the feasibility of evaluating AI-assisted workflows in a research environment that emulates the clinical PET/CT reporting workflow. The findings of this study indicate that the AI-assisted workflow achieved comparable performance to conventional reading but with the potential to increase reporting efficiency without adversely affecting report quality. For experienced PET/CT reporters, this equated to a reduction of almost 20% in scan reading time when using AI assistance, which could translate into significant productivity gains. For example, a 20% improvement in productivity of a senior radiologist reporting complex studies such as PET/CT with an average workload of eight cases per half-day session performed twice weekly for 42 weeks a year equating to 672 cases/year would potentially be able to report an additional 134 cases per year (or 3.35 extra cases per reporting day) with AI assistance. In larger centres with multiple reporters, it is easy to see the economies this could provide, with approximately 17 extra scans reported per 10 half-day sessions/week. Another potential benefit is that an assisted workflow providing automated segmentation could facilitate the quantification of metabolic tumour volume, which is currently not possible to achieve in a busy clinical setting otherwise. Importantly, there were no incidences where AI assistance adversely influenced a reader even when deliberate errors were introduced. One experienced reader had a lower quality score in a single AI-assisted read, which was due to not reporting an incidental ovarian lesion only visible on the CT component. In the context of lymphoma staging under calling stage III or stage IV disease as stage I or limited stage II disease is likely to have the most deleterious effect, because in some high-grade lymphomas, early-stage disease may be treated with a short course of chemotherapy or radiotherapy (21). Reporter confidence was significantly improved by AI assistance, and in the vast majority (>90%) of FP/FN cases, the reporter was able to correctly identify when there was a mistake with the presented segmentation. Trainees were less likely to identify if an area of disease had been missed from the presented segmentations when compared to junior and senior consultant colleagues. This may represent reader fatigue in completing questionnaires or reflect the potential for less experienced reporters to be more vulnerable to AI-derived mistakes.

This is one of the first studies to evaluate the use of AI assistance in the reporting of lymphoma staging PET/CT scans and to look at the influence on speed and report quality. Other studies have assessed the utility of AI assistance in the reporting of oncological CT, brain MRI scans, breast tomosynthesis, MRI spine and prostate-specific membrane antigen PET/CT studies (22–26), all reporting a potential benefit. However, when evaluating AI-augmented workflows, it is essential to understand how tools might influence the user, and if the algorithm needs to be more specific or sensitive in its design. The aim of the present study was to assess this aspect by presenting readers with deliberately flawed segmentations without their knowledge, which still led to a shorter reading duration and did not affect the quality of their reports. However, when asked directly in a questionnaire if the segmentations had been mislabelled, trainees were more likely to be adversely influenced by under-representative segmentations than their consultant colleagues. This may be due to differences in attitudes when completing the questionnaires or reader fatigue, but it does highlight that there is a risk that less experienced readers may be more vulnerable to mistakes introduced by AI assistance. As well as trying to develop tools that protect against this, there also needs to be greater awareness of the risks and benefits of AI tools before routine use in clinical practice and incorporation of these aspects into radiological training (27).

The present study has some limitations. First, a relatively small number of readers and PET/CT cases were used for practical reasons. This initial study was designed to inform a larger trial to further develop and validate a tool for AI-assisted PET/CT reading and reporting in normal clinical practice. However, it is important to note that PET/CT reporting is much more time consuming and complex than other imaging examinations, and that clinical reporting totalled more than 68 h in this study. Second, in this pilot study, the AI-assisted workflow was always the second read, after a standard 6-week washout period following the standard of care PET/CT read and with details of the cases altered, to minimise the chances of the reader remembering the case. However, a stronger study design would be to randomise the standard of care and AI-assisted reads between the two sessions. Third, biases introduced by reporter workstations or scanning protocols were not explored, which is something that is needed to take into consideration when developing PET/CT-based AI software. Finally, the study only focused on measuring the impact of automating a small portion of the clinical workflow with an AI-assisted PET/CT lesion detection and segmentation tool that integrates within the existing clinical workflow. Future studies should investigate the potential benefit of using AI-based technology, such as speed and quality of reporting, as well as patient outcome, through automating other reading and reporting tasks, including the generation of structured reports (2).

## Conclusion

AI reporter assistance in lymphoma staging PET/CT has the potential to improve reader efficiency without negatively affecting quality. These preliminary findings require validation in a larger study in the first instance.

## Data Availability

The raw data supporting the conclusions of this article will be made available by the authors, without undue reservation.
