# Supplementary material for: Comparative effectiveness of standard vs. AI-assisted PET/CT reading workflow for pre-treatment lymphoma staging: a multi-institutional reader study evaluation
Source: Front Nucl Med. 2024 Jan 11;3:1327186. doi: 10.3389/fnume.2023.1327186 (PMC11440880; doi:10.3389/fnume.2023.1327186)
Supplement: Supplementary file 1 [file Datasheet1.pdf]

AI Participant Questionnaire

* Required Information

Reader Assessment

* 1. Case Number

____

2. Which lymph nodes are involved? (please tick all applicable)

- [ ] Cervical neck nodes - unilateral and localised to one area
- [ ] Cervical neck nodes - unilateral and not localised to one area
- [ ] Cervical neck nodes - bilateral
- [ ] Thoracic nodes
- [ ] Abdominal/Pelvis nodes

3. Is there bulky disease (>10cm)? Please provide the location

____

4. Is there splenic involvement? (please tick all aplicable)

- [ ] Splenomegaly
- [ ] Focal lesions
- [ ] Definite splenic involvement
- [ ] Possible splenic involvement

5. Are there sites of extra-lymphatic involvement? (please tick all applicable)

- [ ] Pulmonary
- [ ] Pleural
- [ ] Hepatic
- [ ] Renal
- [ ] Osseous
- [ ] Other (Please specify) __________

6. Were there additional sites of disease not contoured, if so where?

____

7. Was there sites of disease contoured which should not have been, if so where?

8. Where there any significant incidental findings on the PET or CT component?

9. Were you interrupted during your reading of the study?

- [ ] Yes
- [ ] No

10. If so, how long was the interruption?

11. How confident are you that you have identified all sites of disease? **(Select one option)**

1	2	3	4	5	6	7	8	9	10
○	○	○	○	○	○	○	○	○	○
Not confident									Very confident

12. Did the AI tool accurately capture disease extent? **(Select one option)**

1	2	3	4	5	6	7	8	9	10
○	○	○	○	○	○	○	○	○	○
Not at all									Perfectly

Non-AI Participant Questionnaire

* Required Information

Reader Assessment

* 1. Case Number

____

2. Which lymph nodes are involved? (please tick all applicable)

- [ ] Cervical neck nodes - unilateral and localised to one area
- [ ] Cervical neck nodes - unilateral and not localised to one area
- [ ] Cervical neck nodes - bilateral
- [ ] Thoracic nodes
- [ ] Abdominal/Pelvis nodes

3. Is there bulky disease (>10cm)? Please provide the location

____

4. Is there splenic involvement? (please tick all aplicable)

- [ ] Splenomegaly
- [ ] Focal lesions
- [ ] Definite splenic involvement
- [ ] Possible splenic involvement

5. Are there sites of extra-lymphatic involvement? (please tick all applicable)

- [ ] Pulmonary
- [ ] Pleural
- [ ] Hepatic
- [ ] Renal
- [ ] Osseous
- [ ] Other (Please specify) ____________

6. Where there any significant incidental findings on the PET or CT component?

____

7. Were you interrupted during your reading of the study?

- ☐ Yes
- ☐ No

8. If so, how long was the interruption?

____

9. How confident are you that you have identified all sites of disease? **(Select one option)**

1	2	3	4	5	6	7	8	9	10
○	○	○	○	○	○	○	○	○	○
Not confident									Very confident
